# Effect of frailty status on mortality risk among Chinese community-dwelling older adults: a prospective cohort study

**DOI:** 10.1186/s12877-023-03759-8

**Published:** 2023-03-18

**Authors:** Xinxin Zhao, Rui Zhu, Qi Chen, Jia He

**Affiliations:** 1 School of Medicine, Tongji University, 1239 Siping Road, Shanghai, 200092 China; 2 School of Medicine, Shanghai YangZhi Rehabilitation Hospital (Shanghai Sunshine Rehabilitation Center), Tongji University, Shanghai, 200092 China; 3 Department of Health Statistics, Navy Medical University, 800 Xiangyin Road, Shanghai, 200433 China

**Keywords:** Frailty, Mortality, Prediction, Prevention, Older adults

## Abstract

**Background:**

Frailty is associated with mortality among older adults. We aimed to determine the appropriate time and frailty index (FI) threshold for frailty intervention in Chinese community-dwelling older adults.

**Methods:**

In this prospective cohort study, we used data from the 2011 wave of the Chinese Longitudinal Healthy Longevity Study. Follow-up was performed for seven years from baseline. Using the FI to evaluate frailty and define frailty status, we explored the best time point and FI score for frailty intervention, by comparing the relationships of FI and frailty status with mortality.

**Results:**

From 2011 to 2018, 8642 participants were included and followed-up. A total of 4458 participants died during the study period. After adjusting for variables such as age, sex, marital status, education level, and living conditions, the hazard ratio (HR) of mortality risk based on the FI at baseline was 37.484 (95% confidence interval [CI]: 30.217–46.498; *P* < 0.001); female sex, living in the city, being married, and living with spouse were found to be protective factors, whereas ageing was a risk factor for frailty. The mortality risk was higher in pre-frail than in frail participants (HR: 3.588, 95% CI: 3.212–4.009, *P* < 0.001). Piecewise linear regression analysis revealed an FI score threshold of 0.5. When the FI score was > 0.5, the HR of mortality based on the FI was 15.758 (95% CI: 3.656–67.924; *P* < 0.001); when the FI score was ≤ 0.5, the HR of mortality based on the FI was 48.944 (95% CI: 36.162–66.244; *P* < 0.001).

**Conclusion:**

Using FI as a continuous variable to predict death is more accurate than frailty status. The advancement of early interventions for mortality risk reduction is more beneficial in pre-frail than in frail patients, and an FI score of 0.5 was found to be the threshold for mortality prediction using the FI.

## Background

Rapid ageing of the worldwide population has become a major trend in the global demographic structure owing to reduced fertility and increased mortality rates [1, 2]. Frailty is becoming an increasingly obvious and common feature of an ageing older adults; a decline in various physiological functions related to age increases vulnerability to stressors. In addition to disease or disability, frailty is associated with a systemic impairment of physical and cognitive functions, including symptoms, diseases, and life-long deficits [3, 4]. People with frailty are more likely to experience a variety of negative health conditions, such as falls, fractures, hospitalization, need for nursing home placement, disability, poor quality of life, and dementia [5–9].

The frailty index (FI) is one of the most commonly used tools to measure frailty. FI is evaluated based on the concept that frailty is a state caused by a life-long accumulation of health deficits; the higher the number of health deficits, the greater the tendency for frailty. These health deficits include symptoms, disease, disability, abnormal laboratory findings, and social characteristics [10–12]. FI is predictive for adverse outcomes and is directly related to survival outcomes [13–15]. Moreover, compared with chronological age, FI has a stronger correlation with mortality, especially within short intervals less than four years [16].

FI has been shown to vary with time; thus, it is evaluated using cross-sectional studies that cannot accurately predict mortality risk [17, 18]. Therefore, it is necessary to perform mortality risk reassessment using dynamic FI changes [19, 20]. Moreover, frailty is not only associated with age but is also affected by risk factors including impairment of activities of daily living, chronic diseases, depression, poor lifestyle habits, and geriatric syndromes [21, 22]. Effective prevention and treatment can reduce occurrence of frailty in older adults [23]. Hence, mortality risk prediction and early intervention to treat debilitating conditions can prolong survival time, thereby alleviating the pressure on medical care [24].

We aimed to collect and evaluate longitudinal data at different time points, and to accurately determine the best time point for frailty intervention using a long follow-up duration. Our findings will potentially enhance decision-making regarding frailty intervention and the effective utilization of medical resources.

## Methods

### Participants

The Chinese Longitudinal Healthy Longevity Survey (CLHLS) is a nationwide longitudinal survey conducted in a randomly selected half of the counties and cities in 22 of the 31 provinces in China. All the participants provided written informed consent [25]. We used the data from the 2011 wave of the CLHLS, which was followed-up in 2014 and 2018. The medical ethics committee of Tongji University approved this study. Participants were excluded if more than 30% of FI variables were missing or if they died before the 2014 follow-up. Moreover, we excluded individuals who had 80% missing data on cognitive function and less than 30 variables for FI calculation.

### Frailty index

Health deficits were evaluated using the FI. We selected 42 items on self-related health, physical function, psychological and cognitive function, comorbidity, and social deficits [25, 26]. Cognitive function was measured using the Mini-Mental State Examination (MMSE) scale [27]. Binary variables were encoded as 0 or 1. For ordered and continuous variables, encoding was based on the distribution. A score of 2 was assigned if the respondent had suffered from more than one serious disease in the past two years. The FI score was calculated as the ratio of health deficits present to the total number of deficits considered, with values ranging between 0 and 1. Higher scores indicated a higher degree of frailty; FI scores < 0.25 and ≥ 0.25 were considered to indicate non-frailty and frailty statuses, respectively [28, 29]. To find the best intervention site, the non-frailty status is sub-divided into robust and pre-frailty stages according to FI score ≤ 0.1 and 0.1 < FI score < 0.25, respectively.

### Statistical analysis

Cox proportional hazards regression and piecewise linear regression [30] were used to evaluate the relationship between FI and mortality, and the Kaplan–Meier survival function curve was used to estimate the seven-year survival in relation to the FI and frailty status. The areas under the receiver-operating characteristic (ROC) curves (AUCs) of FI and frailty status were calculated to compare the effects of these parameters on death outcomes during the follow-up period. Statistical analyses were performed using SAS version 9.4 (SAS Institute, Cary, NC, USA), IBM SPSS Statistics version 20 (SPSS Inc., Chicago, IL, USA), and R statistical software version 4.2.0 (R Foundation for Statistical Computing, Vienna, Austria).

## Results

A total of 8642 older people participated in the baseline survey in 2011. Table 1 shows the participant characteristics and frailty status at baseline. Participants had a median age of 85.6 ± 11.3 years, with a range of 50–114 years. At baseline, 2020 (23.4%), 2802 (32.4%), and 3820 (44.2%) participants were robust (FI score ≤ 0.1), pre-frail (0.1 < FI score < 0.25), and frail (FI score ≥ 0.25), respectively.

**Table 1:** Participant baseline frailty characteristics

		Robust	Pre-frailty	Frailty	*P* value
Age, *n* (%)					
60–75		955 (11.1)	145 (1.7)	918 (10.6)	< 0.001
76–85		682 (7.9)	464 (5.4)	1193 (13.8)	
86–94		294 (3.4)	852 (9.9)	1074 (12.4)	
95–114		89 (1.0)	1341 (15.5)	635 (7.3)	
Sex, *n* (%)					
Male		1219 (14.1)	865 (10.0)	1821 (21.1)	< 0.001
Female		801 (9.3)	1937 (22.4)	1999 (23.1)	
Residence, *n* (%)					
City		349 (4.0)	526 (6.1)	568 (6.6)	0.001
Town		642 (7.4)	839 (9.7)	1203 (13.9)	
Rural		1029 (11.9)	1437 (16.6)	2049 (23.7)	
Education level, *n* (%)					
Illiterate		748 (8.7)	2074 (24.0)	2218 (25.7)	< 0.001
Primary		871 (10.1)	552 (6.4)	1209 (14.0)	
Middle		351 (4.1)	129 (1.5)	340 (3.9)	
Higher		48 (0.6)	37 (0.4)	48 (0.6)	
Marital status, *n* (%)					
Single		27 (0.3)	18 (0.2)	42 (0.5)	< 0.001
Married		1234 (14.3)	552 (6.4)	1548 (18.0)	
Divorced or widowed		753 (8.7)	2220 (25.8)	2219 (25.8)	
Economic status, *n* (%)					
Poor		147 (1.7)	568 (6.6)	608 (7.1)	< 0.001
Rich		497 (5.8)	387 (4.5)	619 (7.2)	
Middle		1369 (16.0)	1794 (21.0)	2572 (30.0)	
Total 8642		2020 (23.4)	2802 (32.4)	3820 (44.2)	

**Fig. 1:**
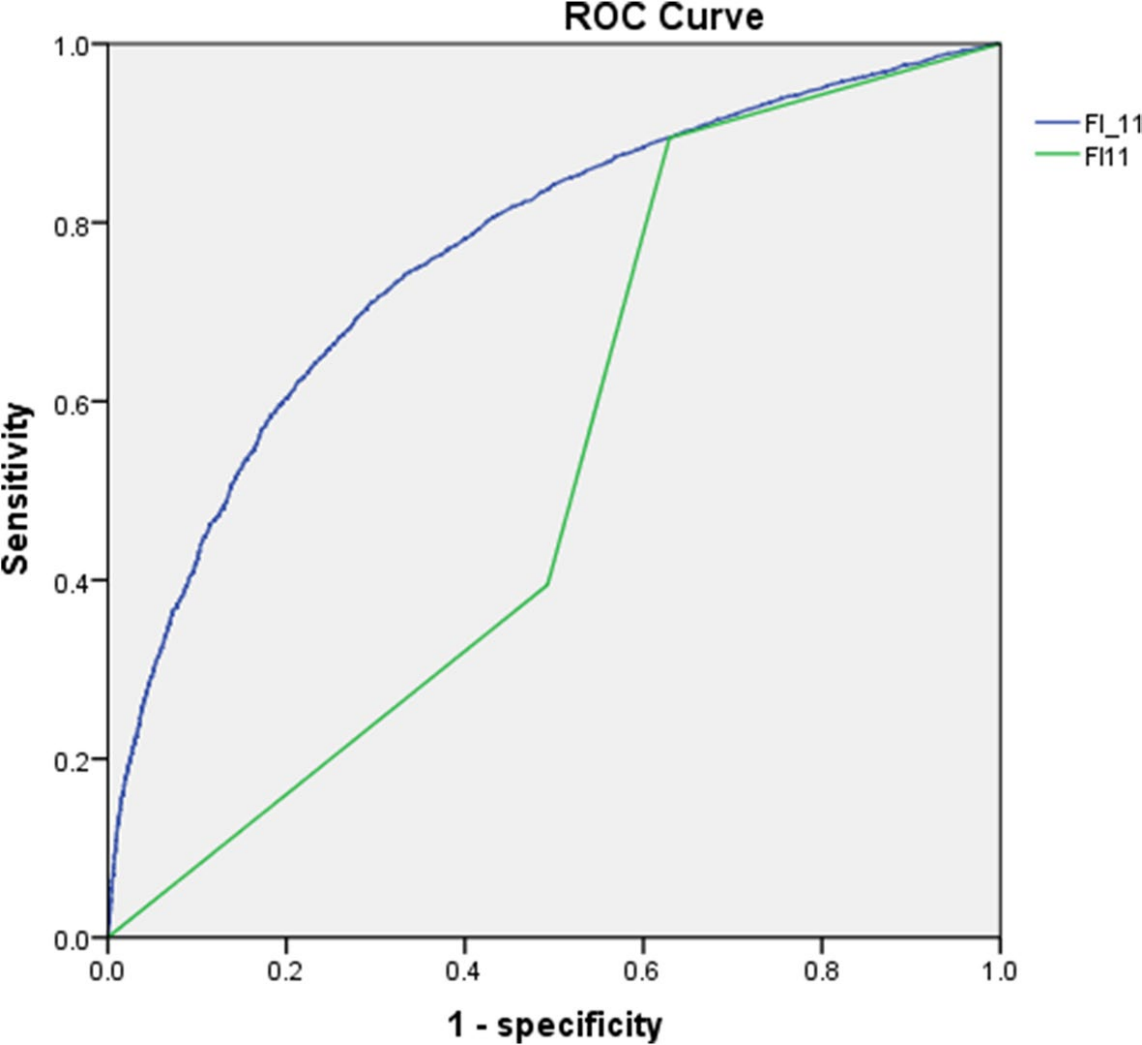
Survival curve of the relationship of frailty and frailty status with mortality Abbreviations: FI_11, frailty index in 2011; FI11, frailty status in 2011

In addition, 4458 participants died during the study period, as observed in 2018. The AUC of FI at baseline was 0.768 (95% CI: 0.758–0.778, *P* < 0.001), whereas the AUC of frailty status was 0.537 (95% CI: 0.524–0.549, *P* < 0.001), thereby showing a weaker prediction with mortality (Fig. 1).

The hazard ratio (HR) of mortality according to the FI at baseline was 37.484 (95% confidence interval [CI]: 30.217–46.498), *P* < 0.001). Female sex (HR: 0.624, 95% CI: 0.584–0.666, *P* < 0.001), living in the city (HR: 0.864, 95% CI: 0.792–0.943, *P* = 0.001), being married and living with spouse (HR: 0.797, 95% CI: 0.736–0.864, *P* < 0.001) were found to be protective factors, whereas ageing (HR: 1.057, 95% CI: 1.053–1.061, *P* < 0.001) was a risk factor for mortality (Table 2).

**Table 2:** Cox regression model analysis of the effect of the frailty index on mortality

	*β*	*SE*	Wald	*P* value	*HR*	95% *CI*	
						Lower	Upper
Age	0.056	0.002	977.972	<0.001	1.057	1.053	1.061
Sex	-0.472	0.033	200.532	<0.001	0.624	0.584	0.666
Residence	-0.146	0.045	10.754	0.001	0.864	0.792	0.943
Marital status	-0.227	0.041	30.716	<0.001	0.797	0.736	0.864
FI_11	3.624	0.110	1086.390	<0.001	37.484	30.217	46.498

Abbreviations: *CI* Confidence interval, *FI_11* Frailty index in 2011, *β* Regression coefficients, *SE* Standard error, *HR* Hazard Ratio

We further classified frailty as non-frailty (FI < 0.25) and frailty (FI ≥ 0.25), and analysed the HR for mortality in different states of frailty. The HR of mortality according to the FI was 2.209 (95% CI: 2.064–2.364, *P* < 0.001) when the frailty status was dichotomized. The female sex, education level, being married, and living with spouse were found to be protective factors, whereas ageing was a risk factor of frailty. The HR for mortality was higher in pre-frail (HR: 3.588, 95% CI: 3.212–4.009, *P* < 0.001) than in frail (HR: 1.820, 95% CI: 1.640–2.021, *P* < 0.001) participants, when the frailty status was evaluated as robust, pre-frailty, and frailty. The female sex, being married, and living with spouse were found to be protective factors, whereas ageing was a risk factor of frailty (Table 3).

**Table 3:** Cox regression model analysis of the effect of frailty status on mortality

	*β*	*SE*	Wald	*P* value	*HR*	95% *CI*	
						Lower	Upper
Non-frailty/Frailty							
Age	0.061	0.002	1148.558	<0.001	1.062	1.059	1.066
Sex	-0.464	0.036	167.825	<0.001	0.628	0.586	0.674
Education level	-0.065	0.026	6.101	0.014	0.937	0.890	0.987
Marital status	-0.202	0.041	24.172	<0.001	0.817	0.754	0.885
Frailty	0.792	0.035	525.386	<0.001	2.209	2.064	2.364
Robust/Pre-frailty/Frailty							
Age	0.058	0.002	1077.900	<0.001	1.060	1.056	1.064
Sex	-0.449	0.033	183.795	<0.001	0.638	0.598	0.681
Marital status	-0.182	0.041	19.724	<0.001	0.834	.769	0.903
Pre-Frailty	1.278	0.057	510.629	<0.001	3.588	3.212	4.009
Frailty	0.599	0.053	126.720	<0.001	1.820	1.640	2.021

Abbreviations: *B* Regression coefficients, *SE* Standard error, *HR* Hazard Ratio, *CI* Confidence interval

**Fig. 2:**
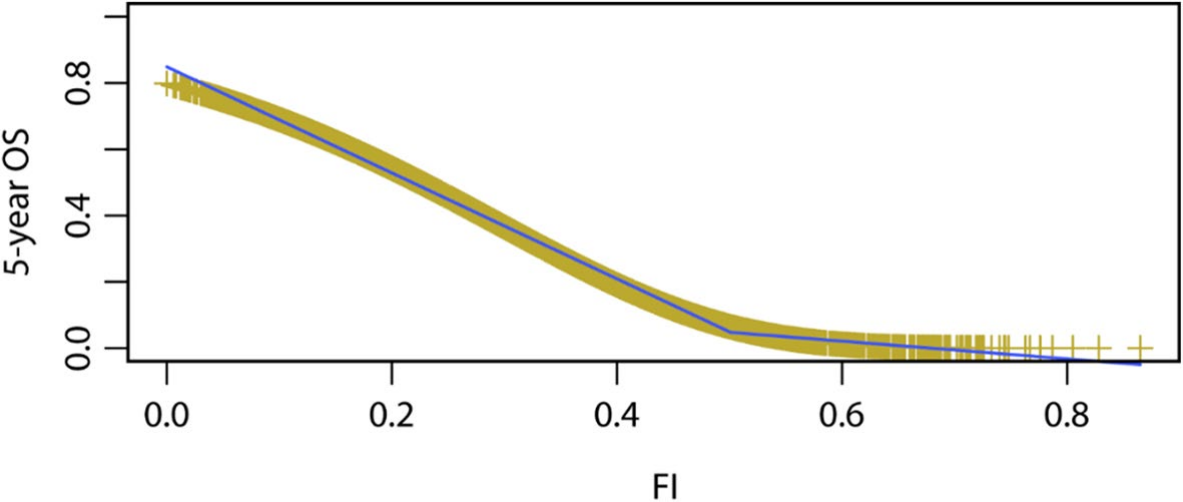
Survival curve of the relationship between frailty and mortality Abbreviations: FI, frailty index in 2011; OS, overall survival

Due to the inconsistency of the different frailty status classifications, we reconsidered FI as a continuous variable. We found that the curves of FI at baseline and seven-year survival rate could be divided into two segments around an FI score of 0.5 (Fig. 2), where the partial regression coefficients were 3.891 and 2.757, respectively. To further explore the effect of a unit increase in FI on the mortality risk, piecewise regression analysis was performed by segment within the FI score ranges between 0–0.5 and 0.5–1. When FI score was > 0.5, the HR of mortality based on FI was 15.758 (95% CI: 3.656–67.924, *P* < 0.001); however, when the FI score was ≤ 0.5, the HR was 48.944 (95% CI: 36.162–66.244, *P* < 0.001). The female sex, living in the city, being married, and living with spouse were found to be protective factors, whereas ageing was a risk factor of frailty (Table 4).

**Table 4:** Piecewise Cox regression model analysis of the effect of frailty on mortality

	*B*	*SE*	Wald	*P* value	*HR*	95% *CI*	
						Lower	Upper
**FI_11 ≤ 0.5**							
Age	0.057	0.002	916.001	<0.001	1.059	1.055	1.063
Sex	-0.491	0.035	197.162	<0.001	0.612	0.571	0.655
Residence	-0.141	0.048	8.806	0.003	0.868	0.791	0.953
Marital status	-0.197	0.043	21.259	<0.001	0.821	0.755	0.893
FI	3.891	0.154	634.752	<0.001	48.944	36.162	66.244
**FI_11 > 0.5**							
Age	0.028	0.006	21.437	<0.001	1.028	1.016	1.041
Sex	-0.242	0.115	4.454	0.035	0.785	0.627	0.983
Marital status	-0.382	0.146	6.789	0.009	0.683	0.512	0.910
FI	2.757	0.745	13.681	<0.001	15.758	3.656	67.924

Abbreviations: *CI* Confidence interval, *FI_11* Frailty index in 2011, *B* Regression coefficients, *SE* Standard error, *HR* Hazard Ratio

## Discussion

Previous studies have investigated the relationship between FI and mortality and predicted the mortality risk based on the static and dynamic FI [20, 26]. However, the relationship between the frailty status and mortality risk has not been studied [31]. To examine the relationship of the FI and frailty status with survival time, we used Kaplan–Meier survival curves to determine whether FI was more strongly associated with mortality than frailty status by calculating the AUCs, and to find that the frailty status was a weaker predictor than using FI with mortality. Previous studies have reported a correlation between FI and short-term mortality; furthermore, our findings demonstrated that FI can be used to predict the seven-year survival rate [21].

Impairment in activities of daily living, chronic diseases, depression, poor lifestyle habits, and geriatric syndromes are risk factors for frailty [32]. Similarly, our study revealed that female sex, living in a city, being married, and living with a spouse are predictive factors of frailty. This is probably attributed to the fact that marital status and living conditions of older adults are related to their mental health and access to medical resources [33]. Previous research had shown a relationship between frailty and type of death; hence, we used survival analysis to evaluate the association between FI and mortality. Our findings provide evidence that clinicians should perform frailty interventions to reduce preventable suffering before death; moreover, these interventions should be performed based on the known risk factors associated with FI [22].

We further explored the relationship between the frailty status and mortality risk at baseline (2011) and during follow-up (2014 and 2018), to establish suitable frailty interventions [29]. When examining the frailty-related mortality risk, we adjusted for demographic (sex and age), and sociological (education level, marital status, and living conditions) factors. When the frailty status was divided into non-frailty and frailty, ageing was considered a risk factor while education level was found to be a protective factor for frailty, in addition to the female sex, being married, and living with spouse. This finding was probably because education increases health literacy. Furthermore, we found that the HR for mortality was higher in pre-frail than in frail individuals. When the FI is > 0.25 as frailty stage, it covers the fraction of FI > 0.5, and thus has less impact on death than pre-frailty stage when the FI is between 0.1 and 0.25. This provides evidence for the possibility of early intervention in pre-frail older adults.

Frailty, defined by phenotype or FI, was found to be significantly associated with an increased risk of all-cause mortality in community-dwelling Chinese older adults based on previous studies [34, 35]. Previous studies showed slightly different results of the relative mortality risk for different frailty levels owing to a lack of a unified frailty classification standard and inconsistencies in frailty status classification [36]. In the present study, we stratified the FI by grade rather than frailty categorization, to perform a more precise risk prediction, and confirm whether 0.5 was the FI threshold. The mortality risk increased with age, and the female sex and being married were found to be protective factors of frailty, which was consistent with previous study findings [37]. Living in the city was found to be a protective factor of frailty when the FI score was < 0.5, indicating that lifespan may be prolonged by exposure to advanced medications in the early state of frailty [38]. When the FI was > 0.5, the effect of frailty on mortality was relatively small because patients with the highest number of health deficits had the highest all-cause mortality rates [26]. A score of 0.5 was the risk threshold when the IF score was close to it, and the risk of death increased significantly with frailty under a score of 0.5.

## Conclusion

Frailty is associated with and predictive of all-cause mortality. Although the effect of intervention in the pre-frailty period may be better than that in the frailty period, intervention with FI below 0.5 may be more beneficial. It is recommended to conduct frailty screening and intervention management for the older adults in Chinese communities.

## Data Availability

The CLHLS analysed during our study are available in the Peking University Open Research Data, [https://opendata.pku.edu.cn/].

## Abbreviations

AUC: Area under the curve
CI: Confidence interval
FI: Frailty index
HR: Hazard ratio
MMSE: Mini-mental status examination
ROC: Receiver-operating characteristic
B: Regression coefficients
SE: Standard error
Df: Degree of freedom
